# Testing that Makes You Think: Development of a Basic Science Test to Promote Future Clinical Learning

**DOI:** 10.1007/s40670-026-02677-9

**Published:** 2026-03-17

**Authors:** Sally Binks, Ryan Brydges, Nicole Woods, Jaimie Coleman, Vyshnave Jeyabalan, Kulamakan Kulasegaram

**Affiliations:** 1 Health Professions Education Research, University of Toronto, 200 Elizabeth Street, 1ES-565, Toronto, M5G 2C4 ON Canada; 2 Temerty Faculty of Medicine, University of Toronto, 200 Elizabeth Street, 1ES-565, Toronto, ON M5G 2C4 Canada; 3 Research Institute, The Institute for Education Research (TIER), 200 Elizabeth Street, 1ES-565, Toronto, ON M5G 2C4 Canada; 4 Temerty Faculty of Medicine, Department of Physical Therapy, University of Toronto, 160-500 University Ave, Room 862, Toronto, ON Canada; 5 Department of Family and Community Medicine, University of Toronto, 500 University Avenue, 5th Floor, Toronto, ON M5G 1V7 Canada

**Keywords:** Medical Education, Formative Testing, Item Development, Response Process Analysis, Distinctive Processing

## Abstract

**Introduction:**

Testing prior knowledge has been found to enhance learning new, related information. In this paper, we report on the development of a multiple-choice question (MCQ) test of basic science knowledge designed to elicit a particular kind of cognitive processing—distinctive processing—that, we argue, may be beneficial to future learning and clinical reasoning. Distinctive processing entails noticing both similarities and differences among concepts or entities. The recruitment and distinctive processing of prior basic science knowledge may optimally prepare learners to understand, remember and apply new, related clinical information.

**Methods:**

We developed two versions of a 19-item basic science MCQ. Both versions of the test had identical stems and correct response options; one version had very similar, plausible or “competitive” incorrect response options while the other version had less similar or “non-competitive” incorrect response options. We hypothesized that the “competitive” test version would elicit more distinctive processing than the “non-competitive” version. We conducted a concurrent verbal protocol to assess the response processes elicited by the two different test versions.

**Results:**

We found that the MCQ items designed with competitive or plausible incorrect answer options elicited more distinctive processing than MCQ items with less competitive or less plausible incorrect response options.

**Discussion:**

We showed that tests can be designed to promote cognitive processes that may be beneficial for future learning and that concurrent verbal protocols can be used to collect response process validity evidence for formative tests.

## Introduction

 Programmatic assessment has been framed as essential to gauge learners’ progress and inform educational strategies in competency-based curricula [1]. At the macroscopic level, programmatic assessment has been operationalized as frequent, low-stakes assessments with meaningful, constructive feedback and coaching [2]. Implementing programmatic assessment, however, has been plagued by issues with translating macro-level assessment changes into direct, in-the-moment benefits to learners [3]. In a case study of the implementation of a programmatic model at Flinders University Medical School, King et al. [4] reported that curriculum developers realized that an emphasis on the analysis of the psychometric properties of items led to a foregrounding of *the assessment-of-learning* function of tests versus their a*ssessment-for-learning* function. Lisk et al. [5], likewise, point to the need for educators to consider how assessments function to influence learners’ cognition to support durable retention, transfer, and preparation for future learning.

Some researchers have turned to the literature on test-enhanced learning for well-supported solutions. *Test-enhanced learning* refers to using testing to enhance, rather than assess or measure, learning through a variety of cognitive, metacognitive, affective and motivational mechanisms [6] The *direct effect* of testing can be harnessed to reinforce memory for studied information simply through retrieval practice; information that is successfully retrieved during a test is more likely to be successfully retrieved in the future [7]. But testing can have effects beyond simply enhancing the retrieval of information. Testing has *indirect effects* that influence both current and future learning. For example, the kinds of cognitive processing evoked when responding to tests may be important in the development of *clinical reasoning* [8, 9]. The foundation of clinical reasoning—and the hallmark of expertise—is a rich, interconnected network of basic science, clinical and experiential knowledge [10–12]. Basic science knowledge functions as a scaffold for clinical knowledge; when basic science concepts serve an explanatory, causal function in relation to clinical phenomena, learners understand, remember and apply new clinical knowledge optimally [13]. Therefore, if the power of testing can be harnessed to, first, activate prior basic science knowledge, and second, to promote the differentiation of that knowledge, learners may be better prepared to learn new, related clinical information.

Researchers often study the indirect benefits of testing by focusing on differing effects of item format (e.g. multiple-choice vs. open-ended). However, evidence suggests that item format matters less than what the item evokes or requires of the learner. In other words, researchers must clarify how the design of tests influence the learning *processes* of trainees [14]. Doing so can better align formative assessment practices with advances in curricula and likely deliver on the promise of test-enhanced learning in advancing assessment for learning and programmatic assessment [15, 16]. In addition to a focus on item format, researchers have, up to now, tended to focus on pre-testing novel, to-be-learned information [17] or on the effects of testing recently acquired information on new learning, for instance, in the word-list learning paradigm [18, 19]. In contrast, the proof-of-concept work we report on is a first step in a program of research that aims to investigate a novel aspect of test-potentiated new learning; that is, the use of testing to activate and elicit particular kinds of cognitive processing of prior, established knowledge in a way that may support interpreting, understanding, remembering and applying new knowledge. Such evidence could expand the validity argument for formative testing as a method of enhancing clinical reasoning [20].

In this paper, we report on the development of a multiple-choice question (MCQ) test of basic science knowledge designed to elicit a particular kind of cognitive processing—*distinctive process*ing—that we argue may benefit future learning. We also report on the accrual of *response process validity evidence* to support the interpretation that the test does, in fact, elicit this type of cognitive processing. Response process validity of formative tests has generally been neglected in higher education [21], and the Health Professions Education (HPE) literatures [22–24].

### Theoretical Background

Response processes are “the mechanisms that underlie what people do, think or feel when interacting with, and responding to, [an] item or task and are responsible for generating observed test score variation” [25]. We propose that *distinctive processing* is a response process that can promote development of clinical reasoning. *Distinctive processing* is the processing of differences in the context of similarity [26]. It consists of two different types of processing—relational and item-specific processing—carried on simultaneously. Relational processing may enhance retrieval by inducing a broad memory search of candidate items; that is, relational processing is memory retrieval at the category or superordinate level. Thus, relational processing promotes the noticing of *similarities*. Item-specific processing (ISP), on the other hand, entails focusing attention on individual items within a category and how their features differentiate them; item-specific processing, then, promotes noticing *differences* [27]. Distinctive processing may promote accurate memory, and likely characterizes expert memory [28]. Multiple-choice test items can be developed to elicit a lesser or greater degree of distinctiveness processing. Items with similar, plausible or competitive response options may elicit relational processing by virtue of their similarity. They may also elicit item-specific processing since, unlike less competitive response options, they compel the test-taker to recruit additional, relevant information from memory to differentiate among them. When response options are competitive, participants perceive some salient conceptual relation(s) that connect response options (relational processing) and then must retrieve specific information that differentiates response options (item-specific processing) in the attempt to select a correct response [29].

### Distinctive Processing and Clinical Reasoning

Reasoning through a differential diagnosis involves distinctive processing as it entails discriminating among manifestations that share some common feature. For example, the complaint of “chest pain” is common to a wide variety of conditions; clinicians must differentiate among them efficiently and accurately using their medical knowledge. As Monteiro et al. [10] state: “…the evidence demonstrates again and again that the essence of expertise is the possession of a *large*,* organised and retrievable body* of both formal and experiential knowledge.” (Italics added). We contend that testing can be designed to induce the kind of distinctive processing that organizes existing knowledge coherently in the learner’s mind, such that new learning is enhanced.

Testing prior knowledge has been found to enhance learning new, semantically or conceptually related information—a forward testing effect—through a variety of mechanisms [30], including priming. First, testing prior knowledge primes attention to important concepts presented in the new, to-be-learned content [31]. Second, testing prior knowledge enhances comprehension of new information and promotes the synthesis and integration of the old with the new [30]. Third, effortful retrieval of prior knowledge through testing with competitive response options causes learners to make more conservative judgments of what they know and thus induces them to optimize their strategies for learning new, related materials, through greater attentional focus and increased time-on-task [8].

### The Current Study

We developed two versions of a 19-item basic science MCQ test and conducted a response process analysis using a concurrent verbal or “think-aloud” protocol. We tested the hypothesis that MCQ items designed with competitive or plausible incorrect answer options induce learners to engage in more distinctive processing than MCQ items with less competitive or less plausible incorrect response options. Our dual aim involves examining whether such tests can promote beneficial processes for learning, and demonstrating how to collect response process validity evidence for formative tests.

## Methods

### Participants and Recruitment

With institutional REB approval, we recruited medical (n=eight) and nursing students (n=eight) from three Canadian universities in the same province who reported having completed the human anatomy and physiology components of their training program. We recruited participants from two professions that share similar anatomical and physiological education content *and* would find the clinical context of our study – intra-aortic ballon counterpulsation – highly relevant. Anecdotally, we knew that the knowledge required in the clinical domain and task that we chose as the context for this study would be similar for nursing and medical students. There was no a priori reason to suppose that that medical students from different Canadian universities would differ in this knowledge or competency given the very similar admissions processes and curricular content used to train students [31, 32]. For experimental balance, we assigned n=four nursing students and n=four medical students to complete the “competitive” version of the basic science MCQ test; and n=four nursing students and n=four medical students to receive the “non-competitive” version. The sample size requirements for think aloud studies vary according to the purpose of the research, its design and the data analysis methods used [33]. For experimental studies, it is recommended that sample size be based on the effect size of the treatment [34]. Differences in distinctive processing elicited by test items may be regarded as a treatment effect; however, we are not aware of any prior studies that report on this phenomenon and, therefore, have no empirical effect size on which to base a sample size calculation. Further, such recommendations refer to treatment effects as indicated by differences in scores by individuals. In this research we are not concerned with comparing individuals but with comparing the different response processes that may be elicited by two versions of a test. The unit of analysis in this work is *utterances* made in response to items/response options, and not participants. Participant scores are not of interest in this work and, therefore, fewer participants are required than might be required for studies for which participant scores are the primary outcome measure [34]. In total, *n* = 16 participants were recruited to participate in the study; the sample of participants yielded 19 test items X 16 participants = 304 responses. For a moderate effect size of d = 0.5, a significance level of α = 0.05, and power (1-β) = 0.8, a sample size of *n* = 44 items are required [33].

Potential participants responded to an email account created for the study; a research assistant coordinated an appointment for a synchronous, one-on-one session for each participant conducted via an online videoconferencing platform. The study materials were hosted on the Qualtrics platform and shared with each participant upon login to the videoconferencing platform. At least one member of the research team was present for every online session in the event of technical difficulties and to provide invigilation.

#### Item Development

We used key concepts from foundational texts on human cardiovascular anatomy and physiology [35, 36] to create a test blueprint that ensured content domain coverage [37]. Each MCQ item consisted of a stem, a correct response option and two incorrect response options or distractors. The difficulty of creating plausible response options increases as the number of distractors increases [38]; since the plausibility of distractors is crucial to the hypothesis of this work, three response options were developed for each item [39]. There was only one correct response for each item; in accordance with best practice guidelines for MCQ item development, there were no “all of the above” or “none of the above” or complex options [38, 40]. Correct response options were presented in random order. Response options were of similar length and grammatical structure. The corresponding items in both versions of the test were administered in the same order.

**Fig. 1:**
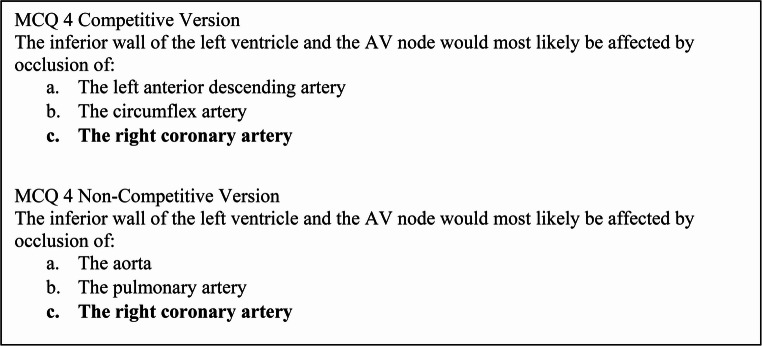
Example of an item from the competitive and non-competitive versions of the test

Distractors were developed using the Key Feature Identification method [38]. This method entails identifying as many key features of the correct response as possible, and then creating distractors by deleting key features for each (for an example, see Appendix A). Thus, distractors having many key features in common with a correct response option are very similar to the correct response option and are likely to be competitive with it (Fig. 1). Distractors for the non-competitive version of the test were developed by deleting all but one or two key features of the correct response option, so distractors were very dissimilar to the correct response. Both versions of the test had identical item stems and identical correct response options; in both versions of the test, the correct response options were in the same position; for example, the correct response option is “c” in the competitive and non-competitive versions of question 4, as shown in Fig. 1. Competitive distractors can also be developed based on common, domain-specific misconceptions of students [38]. A recent publication [41] reporting common misconceptions of medical students about cardiovascular anatomy and physiology was used to develop several items for this test.

Both questions have the same stem; the competitive version (MCQ 4 Competitive Version) has more similar response options (all are coronary arteries) than the non-competitive version (MCQ 4 Non-competitive version) in which all response options are vessels but only one is a coronary artery. The similarity of the competitive response options is assumed to elicit relational processing; the participant must attend to differences among the response options (i.e., engage in Item-specific processing) to select a response.

### Data collection

The facilitator began by sharing the study materials on screen with each participant. In accordance with best practice for the conduct of verbal protocols, the facilitator explicitly identified herself as a domain non-expert to mitigate participants’ potential concerns about being evaluated or judged [34]. The sensation of being evaluated may divert participants’ attention away from the task and toward the facilitator’s reactions, which detracts from the veridicality of the concurrent verbal protocol The facilitator instructed participants to verbalize their thought processes as they worked through the test and informed them that she would provide a non-directive probe to “please remember to say what you are thinking” if they were silent for longer than ten seconds. Once the facilitator provided instructions on thinking aloud, participants worked through three preliminary, “warm-up” general knowledge questions to familiarize themselves with the process and to increase their comfort level with it. The sessions were audio-recorded, transcribed and the transcripts were edited for transcription errors by the principal investigator (SB) who anonymized the transcripts before sending them to a coder.

### Analysis

To analyze response process for the two test versions, two authors, including the PI (SB and JC) independently coded all transcripts. In studies in which the focus is on the content of verbal reports and not on variability among the raters or coders themselves, using only two coders is the accepted standard [33]. The coders are both practicing clinicians with relevant subject matter expertise. We did not blind coders to test version; doing so would have entailed an analysis of utterances without reference to the response options; this would not have constituted a response process analysis. We started with an a priori cognitive model of distinctive processing (Appendix B). To be coded as an instance of distinctive processing, a participant would have to show evidence of noting similarities and differences along at least one relevant anatomical or physiological dimension between or among at least two response options. We also inductively developed codes for specific strategies used by participants in responding to items; these strategies were notable particularly in instances of non distinctive processing. We analyzed the counts of distinctive and non-distinctive processing across both versions of the test, reported as Chi-Squared analyses. Given the mixed population of the study (nursing and medical students), we also used that Mantel-Hanzel Chi-Squared test to test for group differences in the relationship between test type and the codes. We applied all analyses at the level of the multiple-choice item, given that processing type could vary widely across the MCQS in each test for each individual participant. We do not report indices of item difficulty or discrimination because our interest in this work is in evidence for response process validity and not the psychometric properties of the tests. We compared the mean duration in seconds for test completion between groups to determine if findings were at least in part attributable to differences in time-on-task.

### Validity Evidence and Formative Testing

Response process analysis provides “evidence concerning the fit between the detailed nature of performance or response actually engaged in by examinees” [42]. If our findings demonstrate evidence of learners’ cognitive responses to test items, this would support our a priori inferences about how items should, theoretically, tend to elicit particular responses (Appendix B).

## Results

As is accepted practice in response process analysis, we analyzed 20% of the transcripts to gauge the level of agreement between coders, to provide coder training, and to refine code definitions [21]. Transcripts were initially chosen on the basis of the heterogeneity of codes; i.e., those that represented the greatest number of the ten codes. The inter-rater agreement on codes was initially low at 53% to 74%. The code definitions were refined, and several questions that coders disagreed upon were reviewed. Then, a final round of coding was conducted on a randomly selected sample of questions from all transcripts (i.e. questions 4, 8, 12 and 16 from each version of the test). See Appendix C for instances of the coding process. We adopted this strategy because our analysis is at the item or question level and not at the participant level. We calculated the level of agreement between coders for the superordinate categories of distinctive versus non-distinctive processing and non-reasoning processes. We also calculated the level of agreement on coding subcategories; e.g., *guessing* and *recruitment of well-established prior knowledge* as subcategories of *non-reasoning processing*. Ten categories of response emerged from inductive coding, four of which represented some degree of distinctive processing and five categories that represented a reasoning process or some other test-taking strategy that did not entail distinctive processing (Table 1). The remaining category represented the recruitment of well-established knowledge, such that reasoning was unnecessary; thus, data from this category are reported but excluded from the analysis.

**Table 1:** Categories of responses identified through inductive coding of think-aloud transcript data

Response Category	Description
Distinctive Processing	
1. Clear distinctive processing; correct response, correct rationale	Evidence of considering at least two response options and differentiating between them.Selected the correct response and provided acorrect rationale/explanation for the selection.
2. Clear distinctive processing; Correct response, incorrect rationale	Evidence of considering at least two response options and differentiating between them.Selected the correct response but provided anincorrect/incomplete rationale for the selection.
3. Tacit distinctive processing	Evidence that participant was referring to response options, but not articulating thereference.
4. Clear distinctive processing: Incorrect response	Evidence of considering at least two response options and differentiating between them.Selected the incorrect response.
Non-Distinctive Processing	
5. Key term from stem +/- premature closure; i.e., “jumping to a conclusion”	Evidence of focusing on a key term from the question stem and using that term to interpret response options, sometimes leading to premature closure or selecting a response option without dueconsideration of all response options.
6. Marginal knowledge +/- implausible distractors	Evidence of recruitment of marginal knowledge and elimination of implausible distractors; this combination of strategies was used to select a response. Also, verbalizing a sense of familiarity with a response option and being influenced bythat sense of familiarity.
7. Information from stem to reason toward response	Evidence of using information from the question stem to prompt recall and to reason about the answer with minimal or no reference to theresponse options.
8. Test-wise strategies	Evidence of eliminating response options because they differed grammatically or in length from other options or because of the use of terms suchas “always” or “never”.
Non-Reasoning Processes	
9. Guessing	Participant admitted that he/she did not know answer to question and admitted that responseselection was a guess.
10. Recruitment of well-established knowledge	Participant able to provide correct response basedon recall. Did not need to review response options.

### Counts of Distinctive versus Non-distinctive Processing

**Fig. 2:**
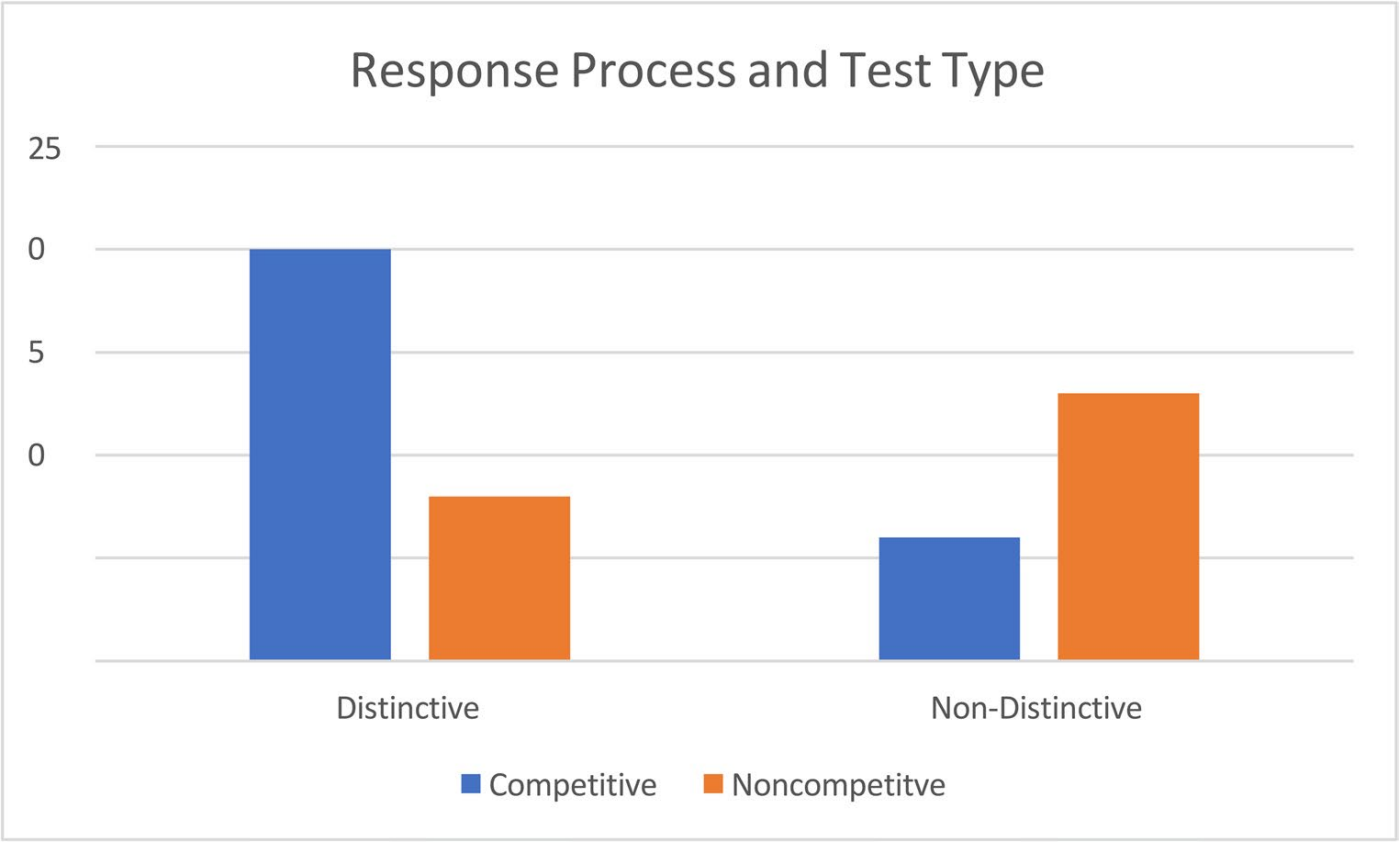
Comparison of counts of distinctive versus non-distinctive codes in competitive versus non-competitive test versions

Counts of instances of distinctive versus non-distinctive processing were made for the random sample of questions (questions 4, 8, 12 and 16) selected from each version of the test. Instances of distinctive processing included categories one through four from Table 1. Instances of non-distinctive processing included categories five through eight from Table 1. Responses that were coded as “non-reasoning processes”—categories nine and ten— or on which raters did not reach agreement were omitted from the analysis. In the competitive version of the test, there were 20 instances of distinctive processing and six instances of non-distinctive processing. In the non-competitive test version, there were eight instances of distinctive processing, and 13 instances of non-distinctive processing (Fig. 2). The competitive version of the test had significantly more instances of distinctive processing than the non-competitive version, and the non-competitive version had significantly more instances of non-distinctive processing than the competitive version (χ^2^ = 7.27, df = 1, *p* = 0.007, φ = 0.7). Post hoc, the power of the test was calculated to be 0.8. The level of coder agreement as to the superordinate code (distinctive processing, non-distinctive processing or non-reasoning processes) was high at 87.5% for both versions of the test. The level of coder agreement for the subordinate codes was, unsurprisingly, somewhat lower (62.5% for the competitive version and 78.1% for the non-competitive test version). 

### Analysis of Group Performance Differences

**Fig. 3:**
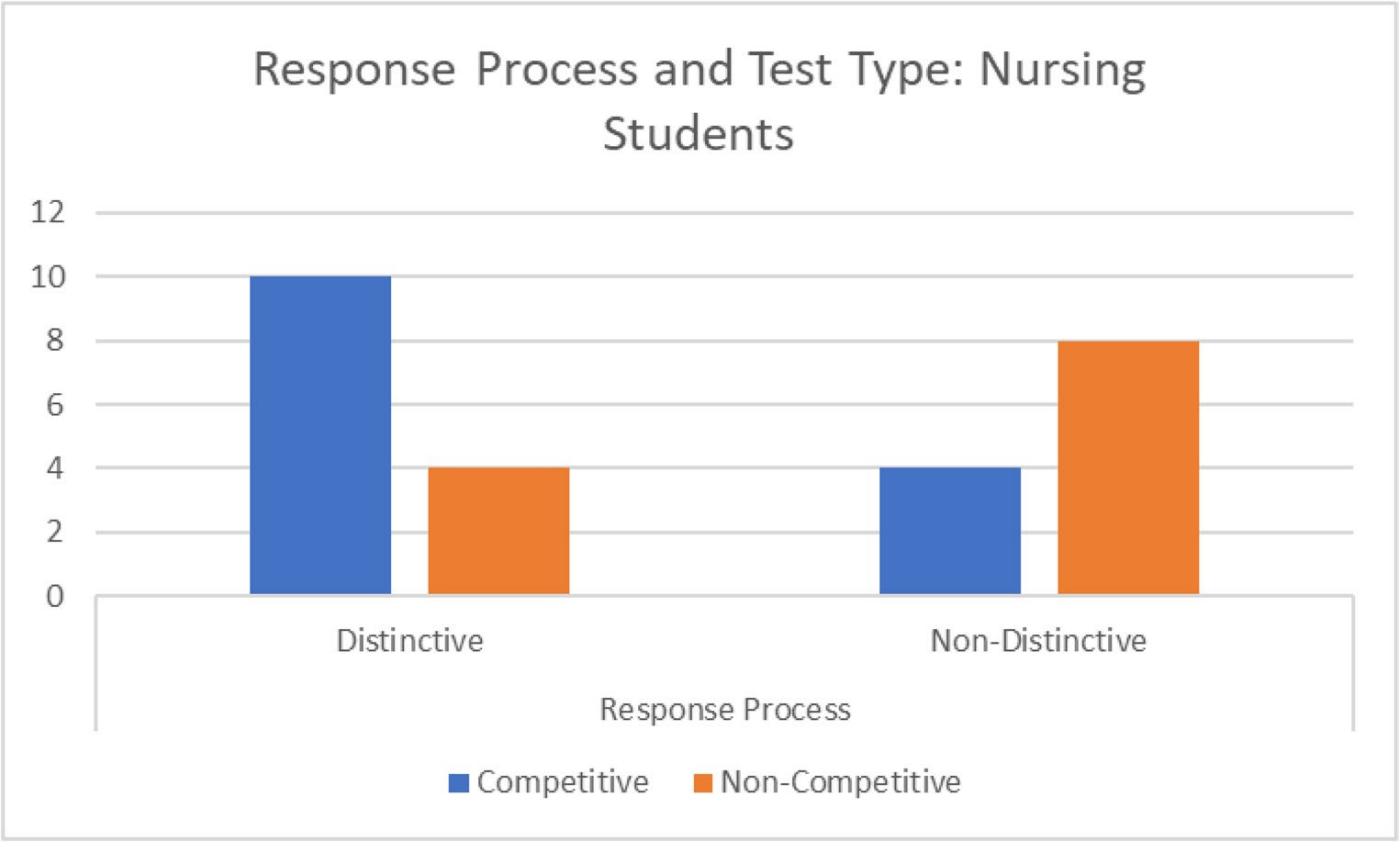
Comparison of counts of distinctive processing versus non-non-distinctive processing codes in competitive versus non-competitive test version for nursing student cohort

**Fig. 4:**
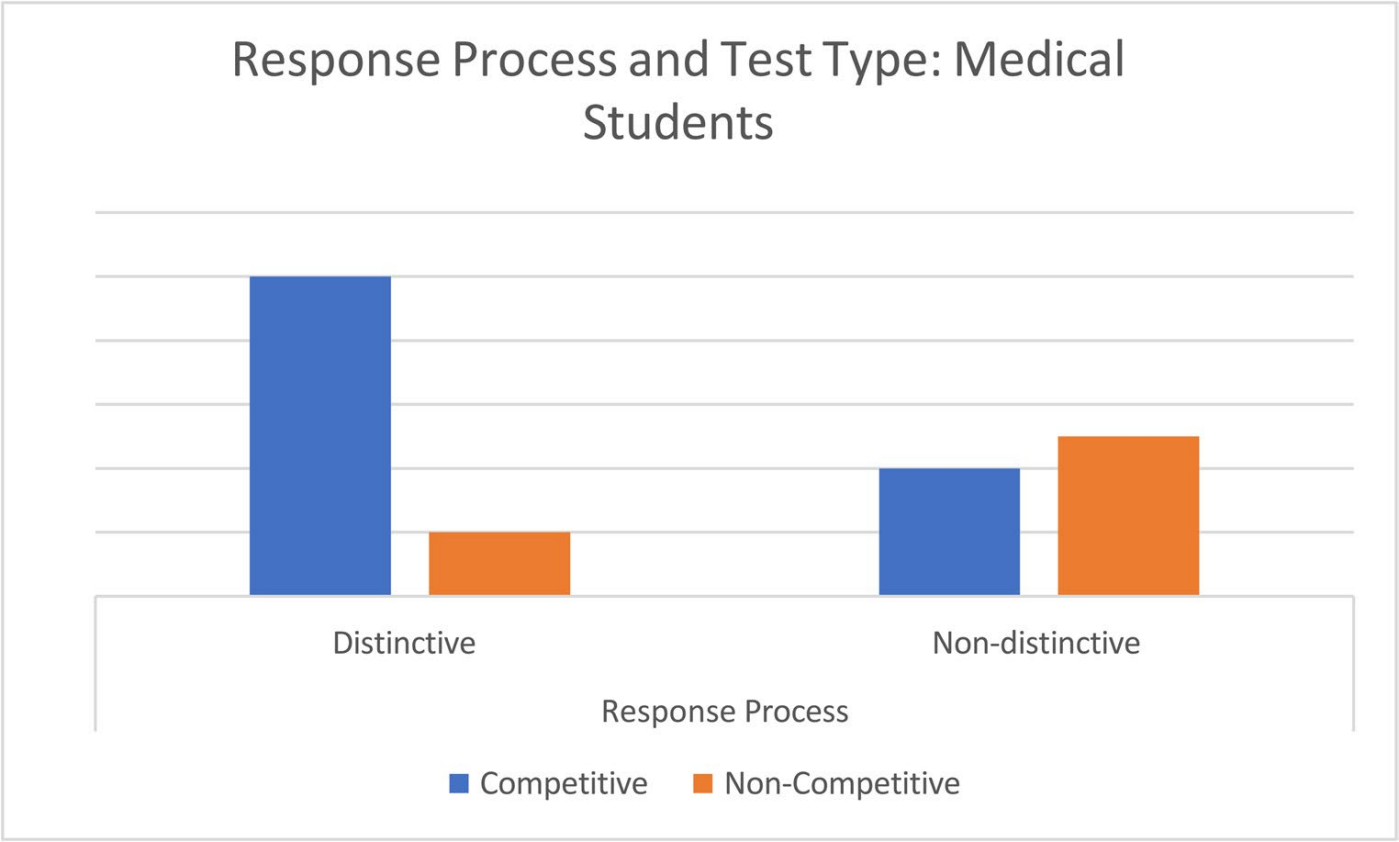
Comparison of counts of distinctive versus non-distinctive processing codes in competitive versus non-competitive test version for medical student cohort

Although the pattern of responses of nursing and medical students were similar between the competitive and non-competitive versions of the test, there was an interaction between the level of response process and test type when stratified by cohort (Figs. 3 and 4). These findings were unanticipated. The medical student cohort had much higher counts of distinctive processing than non-distinctive processing in the competitive version of the test compared to the nursing cohort. In the non-competitive version of the test, the nursing student cohort showed higher levels of non-distinctive processing than distinctive processing compared to the medical student cohort, in which there was little difference in counts of non-distinctive versus distinctive processing [Mantel–Haenszel = 5.47(1), *p* = 0.019; M-H OR = 5.48].

### Time-on-task analysis

We compared time-on-task data across the two test versions to determine if greater time spent on either might have contributed to findings. A Mann-Whitney U test showed no significant difference in time-on-task between the competitive (mdn = 959.5 s) and the non-competitive test versions (mdn = 840 s), U = 36, z = 0.3573, *p* = 0.72, *r* = 0.092.

## Discussion

Our aim involved examining whether multiple-choice question test items designed to have competitive distractors induce more distinctive processing than items with less-competitive distractors. We confirmed our expectation of processing differences between test versions, while demonstrating how to collect response process validity evidence for formative tests. With such evidence, we found that, on occasion, participants did not engage in either type of reasoning process when their prior knowledge was well-established and accessible; there was no need to look at the response options to answer the question correctly. Our results suggest that there may be an interaction between prior knowledge and the capacity to engage in distinctive processing.

Distinctive processing seemed to be engaged mostly when a participant’s prior knowledge was at least somewhat marginal; that is, when item response options triggered recognition and participants recruited prior knowledge that might have been inaccessible otherwise and used this knowledge to differentiate among response options. Conversely, when participants lacked even marginal knowledge, they often use keywords from the questions stem as cues to select their responses. We observed an example of this strategy in how three nursing students in the competitive condition responded to question one (about what produces the dicrotic notch in the arterial pressure waveform). All three participants admitting not knowing what the dicrotic notch is; all used words from the question stem as cues to their responses (two used the word “arterial” as a cue and one used the word “dicrotic” as a cue).

We noted that the responses of some participants showed more—and more elaborated—distinctive processing than that of others. Based on our theoretical model, we suggest that greater benefits of distinctive processing may accrue if learners work to distinguish all three response options in an item rather than focusing on only two of them. That is, they must both recruit and differentiate more prior knowledge on different anatomical or physiologic features. There were instances of distinctive processing in which the participant mentioned and differentiated among all response options very succinctly and could recruit marginal knowledge to answer the question correctly. We found that participants could engage in distinctive processing and select an incorrect response. More concerningly, we also found that participants could engage in distinctive processing and select the correct response but provide an incorrect rationale. These findings raise the concern that distinctive processing could have unintended deleterious consequences if it reinforces faulty knowledge. This concern, however, could be mitigated by providing correct answer feedback [42]. Indeed, incorrect responses offered with high confidence have been shown to be particularly amenable to correct answer feedback, a phenomenon called the hyper-correction effect [43]. Thus, distinctive processing may be of benefit even if it does not lead the learner to the correct response.

We found significant differences in the degree of distinctive processing engaged by nursing versus medical students. One possible explanation for this finding is that some of the concepts represented in the tests—such as some hemodynamic concepts—are not emphasized in undergraduate nursing curricula. Another possible explanation is that medical students are more likely to have completed undergraduate—or even graduate degrees—in health sciences, biology or physiology. Their basic science knowledge, therefore, may be better established than those of most nursing students. As noted, the ability to engage in distinctive processing of prior knowledge probably requires a threshold level of accessible prior knowledge.

Until quite recently, much of the work on test-enhanced learning has focused on the direct or backward effect of testing on memory—the idea that retrieving information makes it more likely that that information will be successfully retrieved in the future [7]. Although researchers have recently become interested in the forward effects of testing or test-potentiated new learning, much of that focus has been on pre-testing, or testing participants on novel information prior to instruction [44, 45]. Our work, in contrast, focuses on testing *prior* knowledge in advance of receiving instruction on new, related information. To a great extent thus far, researchers investigating the forward testing effect have used tasks such as asking participants to learn word lists to examine the mechanisms of test-potentiated new learning. These investigations have identified phenomena such as release from proactive interference, re-set of encoding and event segregation to explain the superior effects of testing over re-study for retention [46]. These findings may prove robust for list-learning, but they may not fully account for memory effects in real-world educational contexts. Retention of new information is optimized when learners understand the structural or conceptual basis of that information. Transfer of new information is optimized when learners are able to distinguish relevant from irrelevant information in the approach to new problems. These two types of processing comprise distinctive processing. We have shown that test items with competitive, similar or plausible response options can elicit distinctive processing to a greater degree than items with non-competitive response options. It is plausible that distinctive processing of their basic science prior knowledge could help learners interpret, understand, remember and apply new, related clinical information.

## Limitations

A limitation of this study is that most of the participants were from a single site; however studies comparing performance on licensing exams suggest that educational outcomes are reasonably consistent across institutions in Canada [32]. We did not anticipate the influence of prior domain knowledge on the capacity to engage in distinctive processing; we believed that participants who had completed their program’s course requirements for human cardiovascular anatomy and physiology would be adequately and equivalently prepared for the experimental task. The finding of significant differences in distinctive processing engaged by nursing versus medical students proved this assumption wrong. However, this finding yielded an important insight: There may be an interaction between knowledge level and the capacity to engage in distinctive processing, and to benefit from distinctive processing of prior knowledge if, indeed, there is any benefit of distinctive processing to future learning. Another limitation of the study is that both versions of the test were created by one author. While the author does have the relevant expertise, further external validation of the appropriateness of the items may have been warranted. Although the primary aim of this study was to determine if competitive test items elicited more distinctive processing than non-competitive items, we left unexplored the assumption that distinctive processing is, in fact, beneficial to future learning. This question will be investigated in future studies.

## Implications and Next Steps

Educators and test developers will likely benefit from our findings that test items with competitive response options appear to elicit higher-order cognitive processing and not merely factual recall. To further explore whether item-specific processing benefits participants’ new learning, future research in this realm could include an assessment of the effect of testing prior knowledge on learning novel material. 

## Conclusions

Test developers have the opportunity to design items to tap particular cognitive processes at particular taxonomic levels. Developers often assume that test takers will respond to test items as intended; however, their assumptions may be wrong [47]. Thus, the lack of response process validity evidence collected during test and item development is a glaring omission in formative and summative assessment. When assessments are intended not to primarily measure but to elicit particular kinds of cognitive processing in formative tests, the requirement for response process validity evidence only increases. With this work, we demonstrated the value of investigating whether test items actually function as they were intended to.
